# Characterization of grain carotenoids in global sorghum germplasm to guide genomics-assisted breeding strategies

**DOI:** 10.1186/s12870-023-04176-0

**Published:** 2023-03-28

**Authors:** Clara Cruet-Burgos, Geoffrey P. Morris, Davina H. Rhodes

**Affiliations:** 1 Department of Horticulture & Landscape Architecture, Colorado State University, Fort Collins, CO 80523 USA; 2 Department of Soil & Crop Science, Colorado State University, Fort Collins, CO 80523 USA

**Keywords:** Sorghum, Carotenoid, Biofortification, Vitamin A, Genomics-assisted breeding, GWAS, Genomic predictions

## Abstract

**Background:**

Crop biofortification is a successful strategy to ameliorate Vitamin A deficiency. Sorghum is a good candidate for vitamin A biofortification, as it is a staple food in regions with high prevalence of vitamin A deficiency. β-carotene—the main provitamin A carotenoid—is below the target concentration in sorghum grain, therefore biofortification breeding is required. Previous studies found evidence that sorghum carotenoid variation is oligogenic, suggesting that marker-assisted selection can be an appropriate biofortification method. However, we hypothesize that sorghum carotenoids have both oligogenic and polygenic components of variation. Genomics-assisted breeding could accelerate breeding efforts, but there exists knowledge gaps in the genetics underlying carotenoid variation, as well as appropriate germplasm to serve as donors.

**Results:**

In this study, we characterized carotenoids in 446 accessions from the sorghum association panel and carotenoid panel using high-performance liquid chromatography, finding high carotenoid accessions not previously identified. Genome-wide association studies conducted with 345 accessions, confirmed that *zeaxanthin epoxidase* is a major gene underlying variation for not only zeaxanthin, but also lutein and β-carotene. High carotenoid lines were found to have limited genetic diversity, and originated predominantly from only one country. Potential novel genetic diversity for carotenoid content was identified through genomic predictions in 2,495 accessions of unexplored germplasm. Oligogenic variation of carotenoids was confirmed, as well as evidence for polygenic variation, suggesting both marker-assisted selection and genomic selection can facilitate breeding efforts.

**Conclusions:**

Sorghum vitamin A biofortification could be beneficial for millions of people who rely on it as a dietary staple. Carotenoid content in sorghum is low, but high heritability suggests that increasing concentrations through breeding is possible. Low genetic diversity among high carotenoid lines might be the main limitation for breeding efforts, therefore further germplasm characterization is needed to assess the feasibility of biofortification breeding. Based on germplasm here evaluated, most countries’ germplasm lacks high carotenoid alleles, thus pre-breeding will be needed. A SNP marker within the *zeaxanthin epoxidase* gene was identified as a good candidate for use in marker-assisted selection. Due to the oligogenic and polygenic variation of sorghum grain carotenoids, both marker-assisted selection and genomic selection can be employed to accelerate breeding efforts.

**Supplementary Information:**

The online version contains supplementary material available at 10.1186/s12870-023-04176-0.

## Background

Carotenoids are the main source of vitamin A in many developing countries where diets are primarily plant based. Cereals provide the majority of calories in developing countries, and most cereal grains accumulate carotenoids —particularly lutein, zeaxanthin, β-carotene, and β-cryptoxanthin [1]. However, the concentration of provitamin A carotenoids —β-carotene, β-cryptoxanthin and α-carotene— is low in cereals compared to fruits, vegetables, and animal derived products. For example, among cereals, maize contains the highest concentrations of carotenoids [1], however, the majority of yellow maize accessions accumulate less than 2 μg/g of provitamin A carotenoids [2], although accessions with higher concentrations have been identified [3]. This concentration is low compared to carotenoid-containing fruits and vegetables, such as carrots (50 μg/g of β-carotene) [4], melon (1 μg/g of β-carotene) [4, 5] and kale (6 μg/g of β-carotene) [6].

Globally, vitamin A deficiency affects an estimated 190 million preschool age children and 19 million pregnant women, contributing to poor growth, intellectual impairment, vision loss, perinatal complications, and increased mortality [7, 8]. Cereal biofortification is one of the most sustainable strategies to combat vitamin A deficiency in developing countries [9]. HarvestPlus has accomplished successful maize vitamin A biofortification through traditional breeding, with current releases in several countries containing β-carotene ranging from 4–16 μg/g [10]. Given the prevalence of vitamin A deficiency in many developing countries and proven success of current biofortified crops, expanding biofortification efforts to other staple crops could significantly reduce global vitamin A deficiencies.

Sorghum [*Sorghum bicolor* (L.) Moench] is a good candidate for biofortification as it is a staple food in regions with high prevalence of vitamin A deficiency, such as in South East Asia and sub-Saharan Africa [11]. Studies have demonstrated that carotenoids are present in sorghum grains, and β-carotene is the main provitamin A carotenoid, with concentrations up to 3.23 μg/g [12, 13, 13–20]. We estimate a sorghum biofortification target value of approximately 12 μg/g β-carotene, although that value will vary depending on the sorghum intake of the target population. Sorghum biofortification using genetic engineering has developed sorghum grain with β-carotene concentrations as high as 12 μg/g [21, 22], but genetically modified sorghum has not been adopted by farmers due to limitations on use of transgenic crops in Africa [23, 24]. However, progress in sorghum carotenoid research suggests that biofortification through breeding is feasible. Genetic studies have demonstrated that there is natural phenotypic variation in sorghum grain carotenoids and that there are genetic components controlling the trait [15, 16, 20]. Genomics-assisted breeding, via marker-assisted selection (MAS) or genomic selection (GS), has the potential to accelerate biofortification efforts by removing the need to employ complex phenotyping methods. Therefore, due to the potential impact of biofortified sorghum in developing countries, the feasibility of sorghum vitamin A biofortification through breeding needs to be tested.

**Fig. 1:**
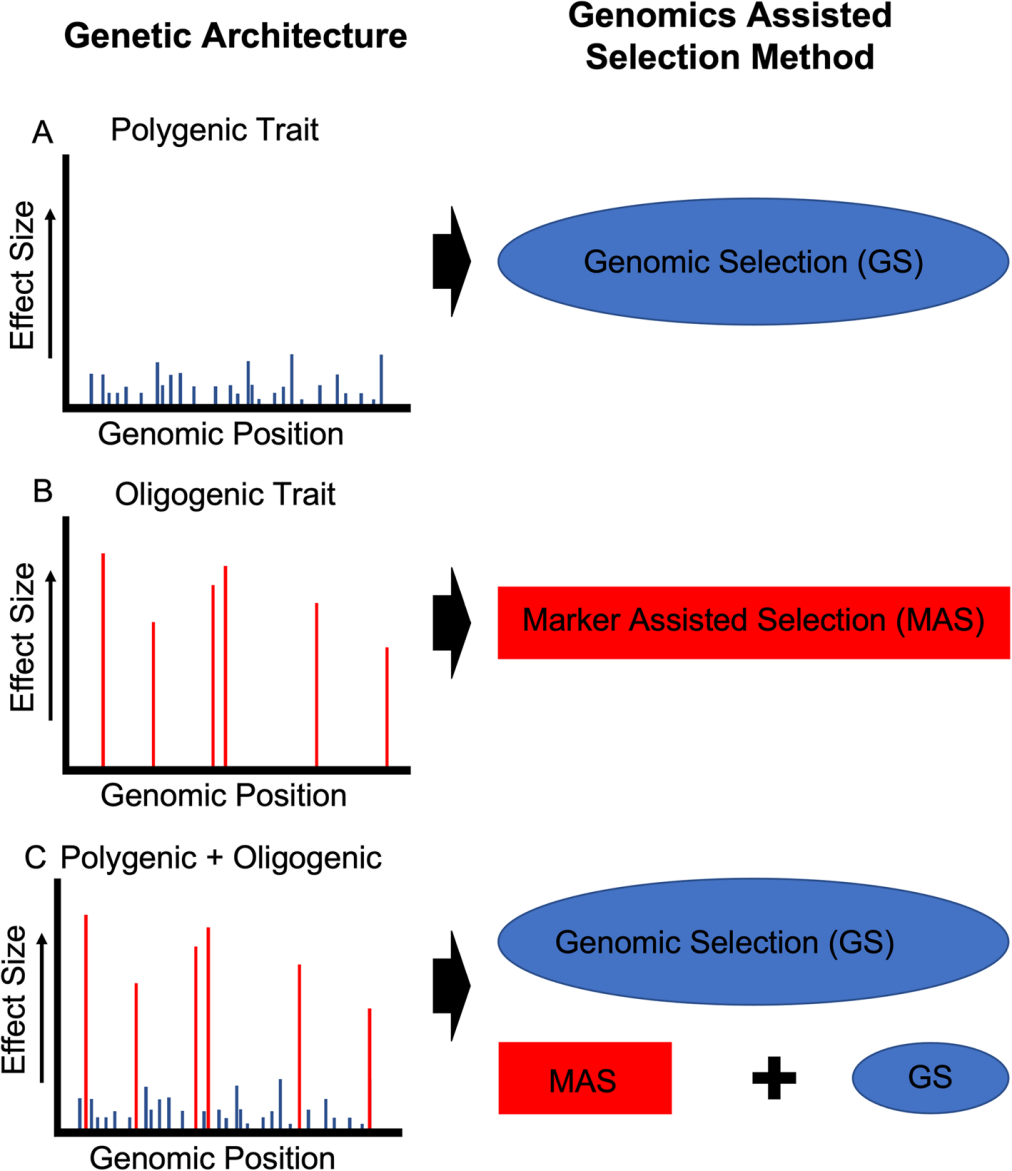
Genomics-assisted selection method based on genetic architecture of a trait. Genomics-assisted selection method for a trait under **A**) polygenic; **B**) oligogenic; and **C**) both polygenic and oligogenic control. Effect size is represented by height of vertical lines; number of genes associated with the trait is represented by number of vertical lines; blue vertical lines represent the polygenic component and red vertical lines represent the oligogenic component contributing to the trait

To use genomics-assisted breeding to develop a carotenoid biofortified sorghum variety, genomic regions associated with variation of provitamin A carotenoids must be identified, as well as efficient selection methods and parental donors. The carotenoid biosynthetic pathway is well understood and conserved in plants [25], facilitating identification of carotenoid genes in sorghum. For β-carotene—the most abundant provitamin A carotenoid in sorghum grain—marker-trait associations have been identified in proximity to *phytoene synthase* (*PSY*), *phytoene desaturase* (*PDS*), and *geranylgeranyl diphosphate synthase* (*GGPS*) genes [15, 16]. For zeaxanthin, marker-trait associations have been identified within *zeaxanthin epoxidase* (*ZEP*), a gene that has also been identified in several other crops as underlying carotenoid variation [26–29]. Previous research in sorghum suggests that carotenoids are oligogenic traits, meaning a moderate number of genes with moderate effect underly the majority of the phenotypic variation detected [15, 16, 20], which is consistent with observations in maize [30] and wheat [31]. The oligogenic variation of carotenoids in cereal grains suggests that MAS may be an effective biofortification method. Alternatively, due to the interconnectedness of carotenoid biosynthesis to other biochemical pathways, a combination of both oligogenic and polygenic models might more accurately explain carotenoid variation. In this instance, GS, or MAS followed by GS, could be employed to simultaneously select for both large and small effect genes (Fig. 1).

Identifying germplasm that harbor alleles for high β-carotene concentrations in sorghum grains is also essential for biofortification breeding. This aspect is perhaps the most challenging, because even though carotenoids are naturally present in sorghum grains, studies have shown that the majority of sorghum varieties have low β-carotene concentrations [14–16, 18, 32]. The limited number of high β-carotene varieties could imply that there is limited genetic diversity for carotenoid concentrations, which would impede gains in breeding efforts. However, the accessions that have been phenotyped for β-carotene concentration is limited, suggesting there could be unexplored germplasm harboring genetic variation. No direct assessment of diversity in high carotenoid sorghum accessions has been conducted, but the high incidence of yellow endosperm from Nigeria in collections and previous studies [16, 33–35] supports that limited diversity is a possibility. Expanding germplasm evaluations and genetic studies could therefore highlight new or conserved genomic regions associated with β-carotene that can be used for MAS, as well as identify a set of parental lines with sufficient genetic diversity to employ in breeding efforts.

Given the potential impact of a high vitamin A sorghum developed through biofortification breeding, and the current gaps in knowledge for sorghum biofortification, in this study we further explored the potential of genomics-enabled breeding tools for increasing carotenoids in sorghum. We hypothesize that both oligogenic and polygenic components of variation exist for sorghum carotenoids (Fig. 1), such that both MAS and GS could accelerate breeding efforts. We expanded the number of sorghum accessions phenotyped for carotenoids, identifying additional high carotenoid accessions, and found evidence for both an oligogenic and polygenic component of variation in sorghum grain carotenoids. We also found that the limited number of known high carotenoid accessions have low genetic diversity among them, but genomic predictions identified new potential donor lines that could harbor novel genetic variation for carotenoids. Lastly, we examined allelic diversity in the *ZEP* gene and found evidence of selection for a high carotenoid allele found in only a few countries.

## Results

### Phenotypic variation of carotenoids in the SAP/CAP collection

To characterize phenotypic variation of carotenoids in sorghum grain, and to confirm previously published phenotype data on one year of samples, we quantified lutein, zeaxanthin, β-carotene, and α-carotene for 446 accessions in the sorghum association and carotenoid panel (SAP/CAP) global collection using high-performance liquid chromatography HPLC (Table S1). Lutein was the most abundant carotenoid, followed by zeaxanthin, β-carotene, and then α-carotene. Raw concentrations for lutein ranged from 0.02–4.61 μg/g, for zeaxanthin from 0.01–2.40 μg/g and for β-carotene from 0.03–1.19 μg/g, with means of 0.58 μg/g, 0.18 μg/g, and 0.17 μg/g, respectively. Α-carotene was detected in only 31 accessions, with values ranging from 0.02–0.11 μg/g. Due to the limited number of accessions with detectable concentrations, α-carotene was omitted from subsequent genetic analysis. High phenotypic correlations were found between β-carotene and zeaxanthin (*r* = 0.74; *p* < 10^–16^), β-carotene and lutein (*r* = 0.78; *p* < 10^–16^), and lutein and zeaxanthin (*r* = 0.75; *p* < 10^–16^). Four accessions, two of which had not previously been phenotyped, had higher concentrations of β-carotene than any accessions previously phenotyped in the SAP/CAP collection.

To account for unbalanced data and accurately predict the genetic merit for carotenoids of the SAP/CAP accessions, best linear unbiased predictors (BLUPs) and heritabilities were calculated for each of the carotenoid traits (Table 1, Table S2). Due to the expected shrinkage effect, lower ranges were obtained for the BLUPs than for the raw concentrations. However, entry-mean basis heritability estimates (Table 1) were high, ranging from 0.78 for β-carotene to 0.92 for zeaxanthin.

**Table 1:** Range, mean, and entry-mean basis heritability (H^2^) for the BLUPs of lutein, zeaxanthin, and β-carotene for the SAP/CAP collection

	**Lutein (μg/g)**	**Zeaxanthin (μg/g)**	**β-carotene (μg/g)**
Minimum	0.15	0.04	0.07
Maximum	3.09	1.83	0.80
Mean	0.58	0.18	0.17
H^2^	0.80	0.92	0.78

### Genome-wide association study of carotenoids in SAP/CAP collection

**Fig. 2:**
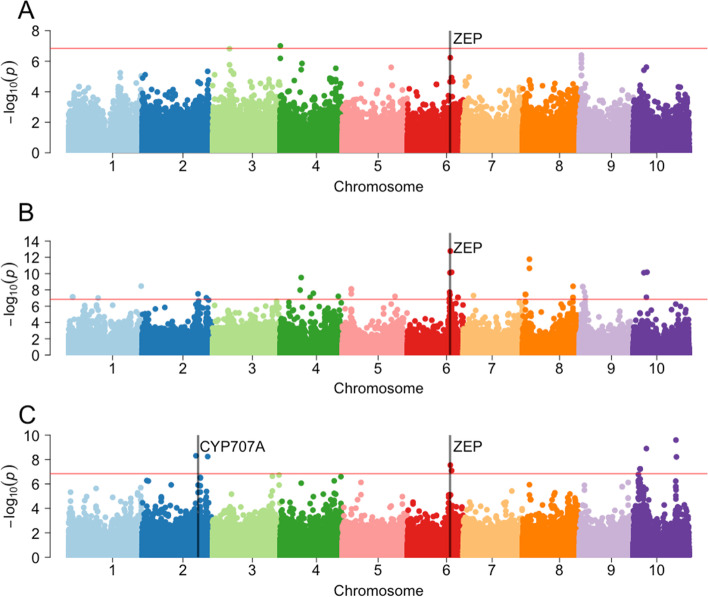
Genome-wide association study of carotenoid BLUP estimates using MLM. Manhattan plot of BLUPs for **A**) lutein, **B**) zeaxanthin, and **C**) β-carotene. The red horizontal line represents the genome wide significance threshold for the Bonferroni multiple comparisons correction at *P* = 0.05

Next, we sought to characterize the genetic architecture of sorghum carotenoids. A previous study [15] suggested that global sorghum grain carotenoid variation is oligogenic, so to further test this hypothesis, we conducted a genome-wide association study (GWAS) using more accessions and replicates. To maximize the number of accessions included, we used BLUPs rather than raw data in order to account for unbalanced data. Marker-trait associations were identified for the BLUPs of the three carotenoid traits evaluated (Fig. 2, Table S3). GWAS was conducted on 345 accessions from the SAP/CAP collection for which we had phenotype and genotype information.

For lutein, only 1 SNP, on chromosome 4 (S04_275231), was above the Bonferroni threshold of significance (Fig. 2A, Table S3). To identify candidates that may not be found using the stricter Bonferroni multiple comparison corrections, we also considered a more liberal False Discovery Rate (FDR) criteria. Under the FDR < 0.05 threshold, 7 significant single nucleotide polymorphisms (SNPs) were identified, corresponding to four regions of association on chromosomes 3, 4, 6 and 9. Three of these SNPs were located in a region around 2.17 Mb on chromosome 9, which is not near any a priori candidate genes. The only association in proximity to an a priori candidate gene was at SNP S6_47123508, near Sobic.006G097500 (401 kb away), an a priori candidate gene that is annotated as a putative ortholog of the maize *ZEP* gene.

Zeaxanthin had the highest number of marker-trait associations above the Bonferroni significance threshold, with 39 significant SNPs in 17 regions across all chromosomes except chromosome 3 (Fig. 2B, Table S3). The most prominent association was on chromosome 6 between 45.9–48.6 Mb, with six significant SNPs, three of which were among the top ten most significant associations. The most significant association for zeaxanthin was the SNP near the *ZEP* gene (S6_47123508; 401 kb away) that was also associated with lutein. There was also an association on chromosome 2 (S2_61694864), which is in proximity to Sobic.002G225400 (42 kb away), an a priori candidate gene annotated as an *abscisic acid 8'-hydroxylase 3* (*CYP707A*).β-carotene had ten significant marker-trait associations for a total of six regions of association across chromosomes 2, 6 and 10 (Fig. 2C, Table S3). Chromosome 10 had the highest number of marker-trait associations, particularly within a region around 7.48 Mb. There was also a SNP on chromosome 10 (S10_14377366) that was significantly associated with both β-carotene and zeaxanthin, which is not in proximity to any a priori candidate genes. Among the ten markers associated with β-carotene, only SNP S06_47123508, 401 kb from the *ZEP* gene, was in proximity to an a priori candidate gene.

### Genetic relationship of sorghum carotenoids in SAP/CAP global collection

**Fig. 3:**
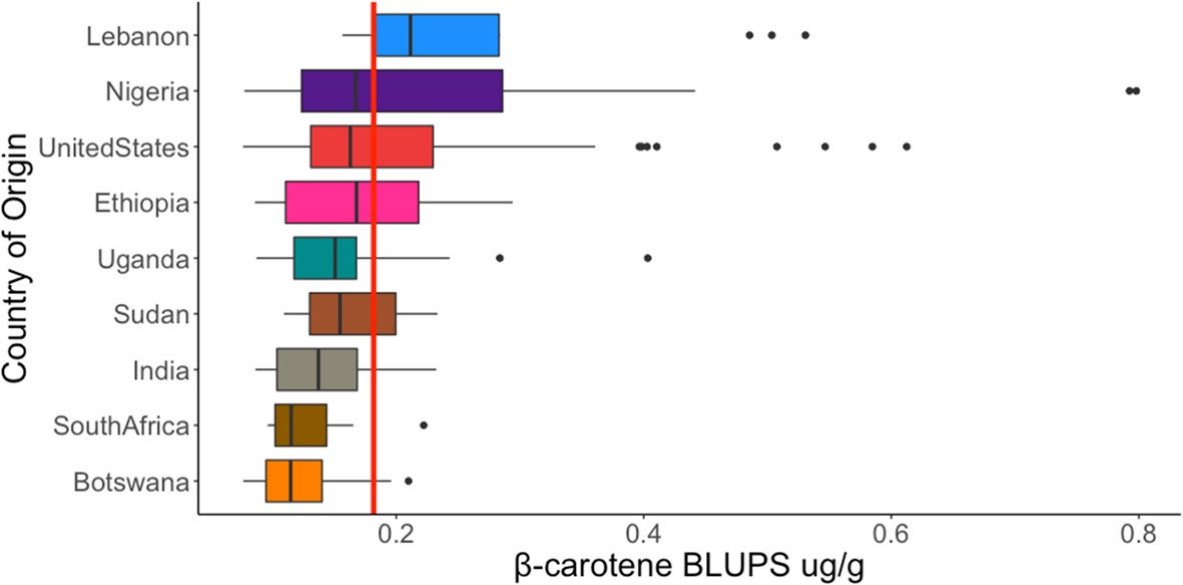
Distribution of β-carotene BLUPs among countries in the SAP/CAP collection. The red vertical line represents the SAP/CAP average across all accessions. Countries with less than 8 accessions were excluded

Next, we tested if carotenoid variation is structured by genetic relationship and geographic origin. Since provitamin A carotenoids are our primary target, we focused on β-carotene concentrations for this analysis. Country of origin was obtained from the USDA NPGS GRIN database for the accessions in the SAP/CAP collection. Countries that had less than eight accessions were discarded from the analysis. In the SAP/CAP collection there were nine countries represented by more than eight accessions: Botswana, Ethiopia, India, Lebanon, Nigeria, South Africa, Sudan, Uganda, and the United States. β-carotene BLUP estimates among this subset of 309 accessions had the same range and average as the full set of SAP/CAP global collection (Table 1, Fig. 3). Interestingly, accessions from most of the countries had average values below the global average for β-carotene of 0.17 μg/g (Fig. 3). Furthermore, β-carotene distribution for the accessions of Sudan, South Africa, India, and Botswana were almost completely below the global average. In contrast, accessions from Lebanon had the highest average β-carotene BLUPs estimates with the majority of their accessions above the global average. Notably, Nigeria, had the widest range of variation as well as the highest β-carotene concentrations among the countries.

**Fig. 4:**
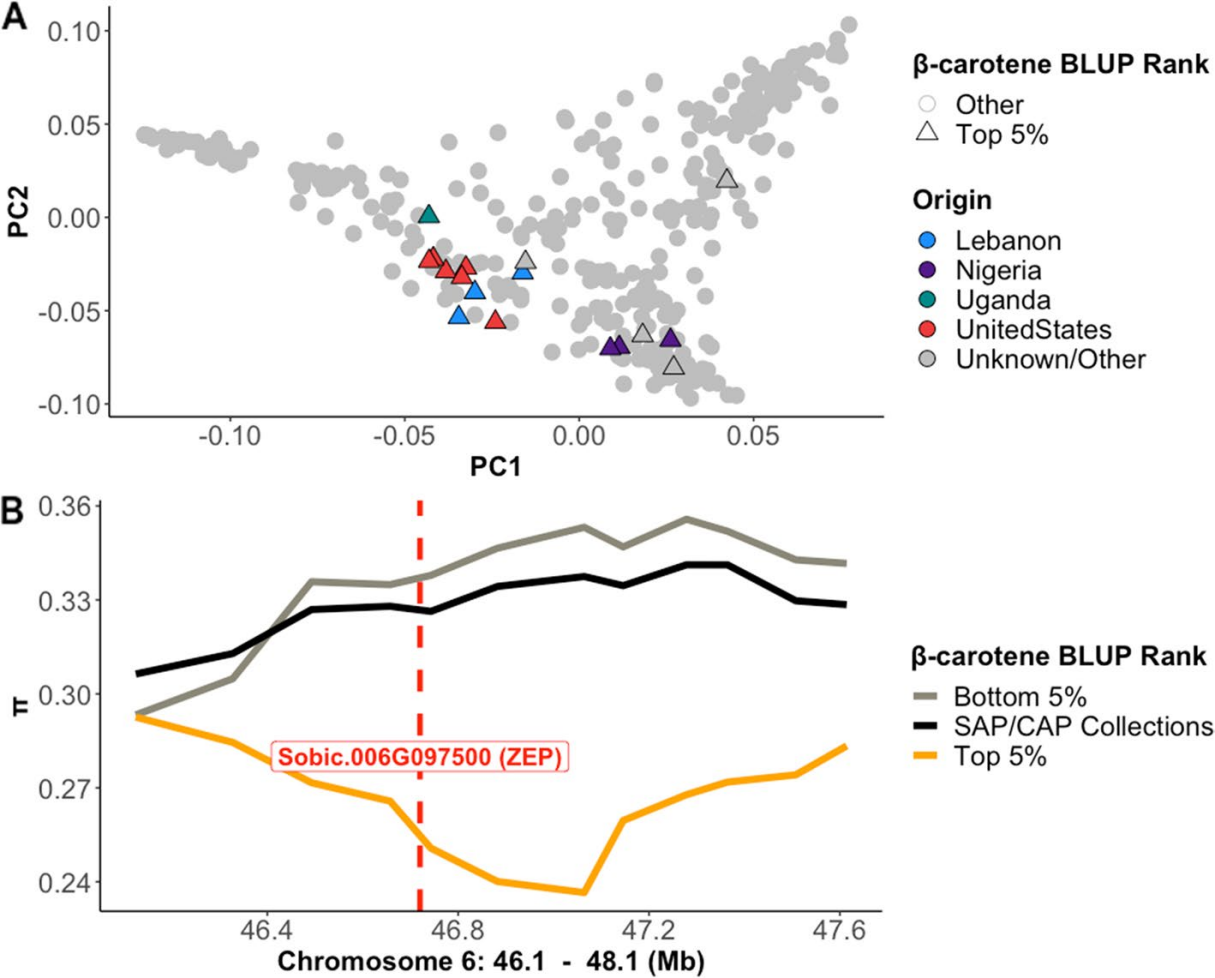
Additive genetic relationship of SAP/CAP accessions based on the top 5% and bottom 5% rankings for β-carotene BLUP estimates and allelic diversity surrounding marker trait association. **A** Accessions plotted according to the first two principal components for sorghum kinships coded by country of origin and ranking for β-carotene BLUP estimates. **B** Nucleotide diversity of the region 1 Mb upstream and downstream of marker S06_47123508. The gray and orange lines represent nucleotide diversity for the bottom and top 5% rankings for β-carotene BLUP estimates, respectively. The black line represents the nucleotide diversity for all of the accessions in the SAP/CAP collections. The red dashed line represents the start position for the *ZEP* gene

Based on the limited geographic distribution of high carotenoid sorghums, we hypothesized that the high carotenoid germplasm originates from a narrow genetic pool. To test this hypothesis, we conducted a principal component analysis (PCA) to evaluate the genetic diversity of the high carotenoid accessions identified in the SAP/CAP collection. The high carotenoid accessions were defined as those within the top 5% for β-carotene BLUP estimates, which consisted of 19 accessions ranging from 0.40 to 0.80 μg/g β-carotene. The majority of the high carotenoid accessions originated in the United States (8 accessions), followed by Nigeria (3 accessions) and Lebanon (3 accessions) (Fig. 4A). Interestingly, the three high carotenoid accessions from Nigeria grouped together and were clustered separately from most of the other high carotenoid accessions, and another high accession of unknown origin did not group with any other high carotenoid accessions, suggesting three genetically distinct high carotenoid groups (Fig. 4A).

To further test our hypothesis on a narrow genetic pool for high carotenoid lines, we evaluated the genetic diversity surrounding the most prominent SNP identified by GWAS for all three carotenoids (S06_47123508). We analyzed a window of 1 Mb upstream and downstream of S06_47123508, which encompassed 1,665 SNPs. Nucleotide diversity was decreased in the high carotenoid accessions, but not in the low carotenoid accessions (defined as the lowest 5% for β-carotene BLUP estimates) or in the complete set of SAP/CAP collection accessions. The most prominent region of low nucleotide diversity was surrounding SNP S06_47123508, a region which includes the a priori candidate gene encoding *ZEP* (Fig. 4B).

### Prediction of carotenoid breeding values in unexplored germplasm collection

Next, we sought to explore if there exists unidentified high carotenoid germplasm in additional germplasm collections. Publicly available genotype data was obtained for germplasm collections from six countries: Ethiopia, Haiti, Niger, Nigeria, Senegal, and Sudan. Together with the SAP/CAP collection, the dataset consisted of 60,129 common SNPs with less than 20% of data missing for 2,488 accessions. There were 361 accessions from Ethiopia, 296 from Haiti, 516 from Niger, 180 from Nigeria, 420 from Senegal, 319 from Sudan, and 396 from the SAP/CAP collection. Most of this germplasm is photoperiod sensitive making it difficult to phenotype in temperate regions such as the United States. For example, among the 396 accessions from the SAP/CAP collection for which we had genotype information, we were only able to phenotype the 345 accessions that were photoperiod sensitive. The inclusion of germplasm from Haiti’s breeding program served to generate hypotheses on the best biofortification approach for the program, providing guidance to the breeder by indicating if there is currently promising germplasm in the program or if donor lines must be introduced to introgress high carotenoid alleles.

**Fig. 5:**
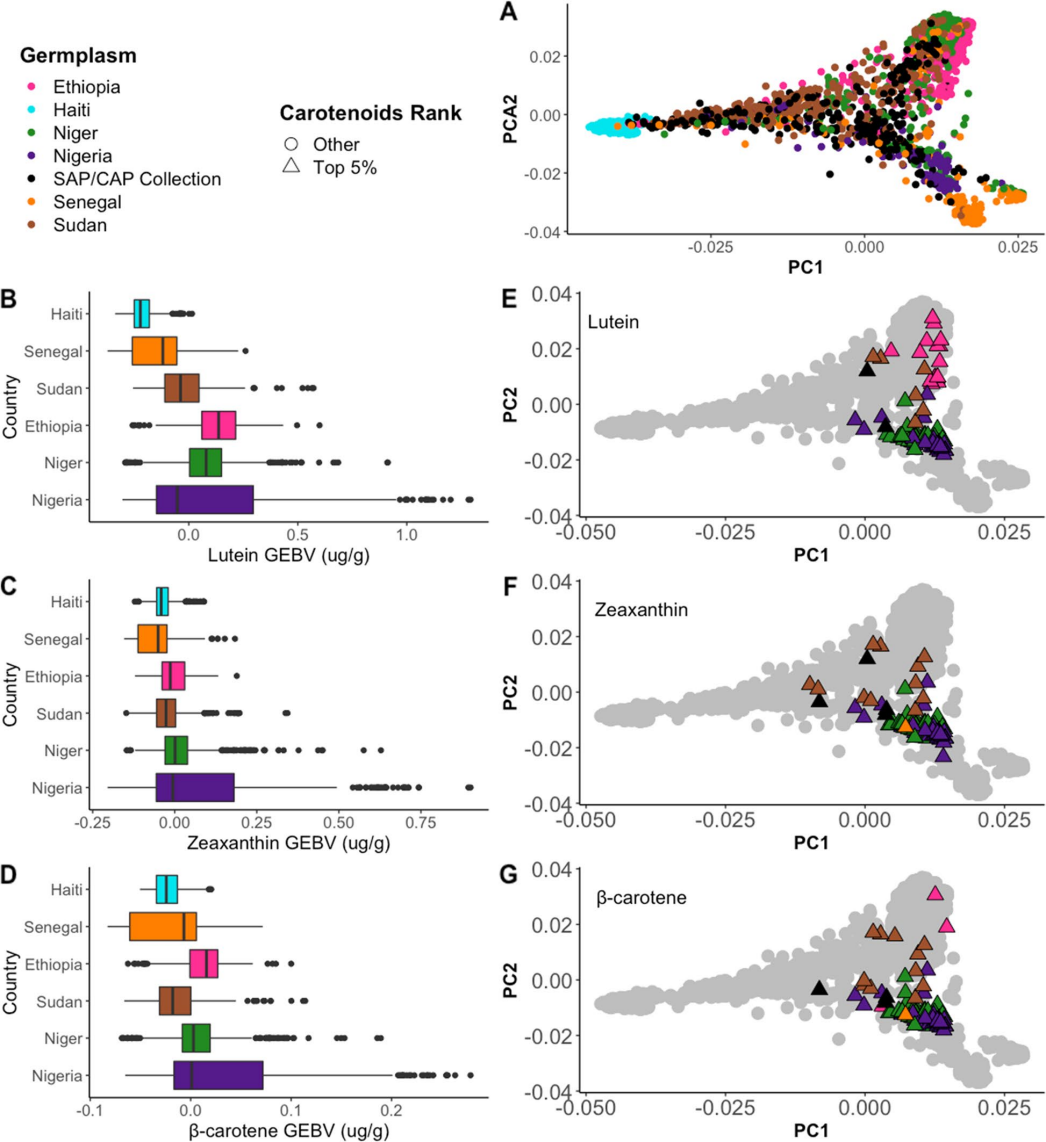
Carotenoids in unexplored germplasm collections. **A** PCA of additive genetic relationship for unexplored germplasm collections and SAP/CAP collection; boxplot of distribution of GEBVs aggregated by country and ordered by lowest to highest carotenoid for **B**) lutein, **C**) zeaxanthin, and **D**) β-carotene; PCA of the genetic relationships of the top 5% of **E**) lutein, **F**) zeaxanthin, and **G**) β-carotene GEBVs in the unexplored germplasm collections

Genomic prediction has the potential to guide resource allocations by identifying the most promising germplasm to test in future work. We first explored the feasibility of the SAP/CAP collection as a training population for the unexplored germplasm collections. For this, the genetic relationship among the unexplored germplasm collections and the SAP/CAP collection was tested with a PCA, highlighting the country of origin for each accession (Fig. 5A, Fig. S2). Based on the scattered distribution observed and the presence of accessions across PCA clusters, the SAP/CAP germplasm collection is an appropriate training population (Fig. S2). Germplasm from Haiti, Ethiopia, Niger, Nigeria, and Senegal formed independent clusters, indicating genetic similarities within but not between countries. Haiti segregated the most distantly, followed by more sparsely grouped germplasm from Senegal and Nigeria. The distant genetic relationship of Haitian germplasm with the other countries was expected as these materials are from a breeding program that went through a recent bottleneck after a sugarcane aphid infestation [36]. Germplasm from Niger and Ethiopia clustered very close together, but separate from the other countries. As expected based on previous studies [37, 38], accessions from the Sudan collection and SAP/CAP collection were scattered across all clusters, rather than clustering together, indicating high genetic diversity. The scattered distribution of the SAP/CAP collection confirms that it is an appropriate training population for genomic predictions in the unexplored germplasm.

Next, we estimated genomic estimated breeding values (GEBV) from the BLUPs of β-carotene, lutein, and zeaxanthin in the unexplored germplasm collections and the SAP/CAP collection (Table S4). Lutein GEBV ranged from -0.37 to 2.16 μg/g, with an average prediction accuracy of 0.62 and a genomic heritability of 0.96 (Table 2). For zeaxanthin, GEBV ranged from -0.20 to 1.44 μg/g, with a prediction accuracy of 0.69 and a genomic heritability of 1.00 (Table 2). Lastly, for β-carotene, GEBV values ranged from -0.08 to 0.46 μg/g, with a prediction accuracy of 0.67 and a genomic heritability of 0.75 (Table 2). Interestingly, there were no accessions in the unexplored germplasm that had predicted GEBV for β-carotene higher than the highest values in the SAP/CAP collection, however there were some accessions that had values among the highest in all the collections (Fig. S1 and Table S4). Finally, as seen in the SAP/CAP accessions, high correlations were identified for GEBV between β-carotene and zeaxanthin (*r* = 0.89; *p* < 10^–16^), β-carotene and lutein (*r* = 0.87; *p* < 10^–16^), and lutein and zeaxanthin (*r* = 0.85; *p* < 10^–16^).

**Table 2:** Range of GEBV, average prediction accuracy and genomic heritability (H^2^) for the lutein, zeaxanthin, and β-carotene for the unexplored germplasm collections and SAP/CAP collection

	**GEBV Lutein (μg/g)**	**GEBV Zeaxanthin (μg/g)**	**GEBV β-carotene (μg/g)**
Minimum	-0.37	-0.20	-0.08
Maximum	2.16	1.44	0.46
Average Prediction Accuracy	0.62	0.69	0.67
H^2^	0.96	1.00	0.75

To further explore geographic patterns of sorghum carotenoid distribution beyond the SAP/CAP collection, we aggregated GEBV by country using the unexplored germplasm collections (Fig. 5B-D). Nigeria had the highest GEBV and range of values for all three carotenoids, followed by Niger. In contrast, Haiti had some of the smallest carotenoid GEBV values, as well as the smallest range of values. Interestingly, Ethiopia had several accessions with high GEBV for lutein, but only three high accessions for β-carotene, and no high accessions for zeaxanthin. Similarly, Senegal had one accession with a high GEBV for β-carotene and zeaxanthin, but not for lutein. These differences suggest that although the three carotenoids are highly correlated—consistent with common genetic controls—there are independent genetic controls, as well.

Next, we looked at the genetic relationships among the predicted top 5% for each carotenoid using a PCA for the GEBV (Fig. 5E-G, Table S5). The pattern of distribution differed by carotenoids, but the majority of the accessions were clustered by country. Lutein (Fig. 5E) had two major clusters corresponding to Ethiopian accessions and a combination of accessions mostly from Nigeria and Niger. For zeaxanthin (Fig. 5F) and β-carotene (Fig. 5G), the clustering was similar, with the accessions of Nigeria and Niger forming the tightest cluster. Accessions from Sudan and the SAP/CAP germplasm were scattered for the three carotenoids, suggesting they are genetically distinct. Taken together, a proportion of accessions predominantly from Nigeria and Niger formed the most distinct cluster in the PCA for the three carotenoids, indicating they are genetically similar. The accessions with the highest GEBV for β-carotene were also part of this cluster.

### Allelic diversity and geographic distribution of ZEP allele

To further test the hypothesis that high carotenoid lines originate from a narrow genetic pool, we analyzed the SNPs inside the *ZEP* gene in the SAP/CAP collection and unexplored germplasm collections. In the SAP/CAP collection, we identified 14 SNPs in the *ZEP* gene with minor allele frequency (MAF) > 0.05. Due to low marker density, the majority of these SNPs were absent in the unexplored germplasm collections. However, SNP S06_46717975 was present in the SAP/CAP collection and the SNP data set for Haiti, Niger, and Nigeria germplasm (Table S6). This SNP is found within the *ZEP* gene and was previously identified by our group as associated with zeaxanthin variation [15]. S06_46717975 was found to be bi-allelic with A/G variants present among the germplasm. The minor allele ‘A’ is moderately common globally, with 10% presence in SAP/CAP collection. However, among countries there are striking differences in the allele frequency; for instance, 24% in Nigeria versus 2% in Niger and 0% in Haiti germplasm.

**Fig. 6:**
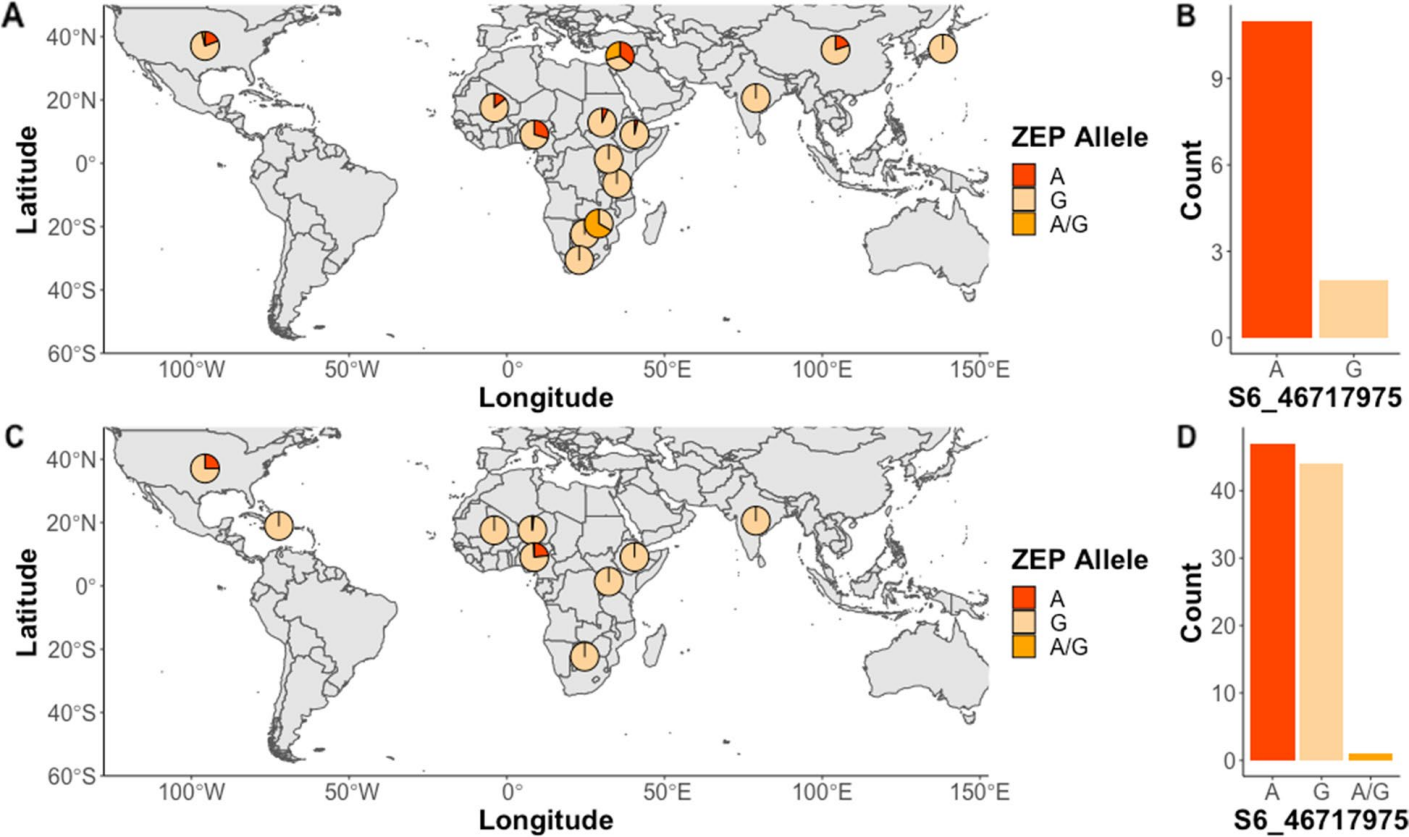
Geographic distribution of the SNP S06_46717975 inside the *ZEP* gene and the distribution of allelic classes in high carotenoid germplasm. **A** Geographic distribution for accessions in the SAP/CAP collection. **B** Distribution of allelic classes for the top 5% rankings for β-carotene BLUP estimates. **C** Geographic distribution for accessions in unexplored germplasm collections and the SAP/CAP accessions without phenotype; **D** Distribution of allelic classes for the top 5% rankings for β-carotene GEBV where A is the minor allele

We next explored if there were any patterns between allelic variant, geographic distribution, and carotenoid content (Fig. 6). In the SAP/CAP collection, there was a correlation between allelic type and country of origin with the United States, Lebanon, and Nigeria, the countries with the highest prevalence of the ‘A’ allele (Fig. 6A). Among the high carotenoid accessions in the SAP/CAP collection (defined as the top 5% for β-carotene concentration), the ‘A’ allele was present in 85% of them (Fig. 6B). We then analyzed the alleles in the unexplored germplasm collections and the SAP/CAP accessions that were not phenotyped. Similar patterns were observed for the geographic distribution of the ‘A’ allele with the highest prevalence in the United States and Nigeria (Fig. 6C). Surprisingly, the difference in the distribution of the ‘A’ and ‘G’ alleles was not nearly as pronounced in the predicted high carotenoid lines based on β-carotene GEBV (Fig. 6D).

## Discussion

### Genetic diversity among high carotenoid lines

A vitamin biofortified sorghum variety has the potential to positively impact the livelihood of millions that rely on it as a dietary staple. We estimate a β-carotene target value of 12 μg/g for biofortified sorghum grain. Although the highest β-carotene content measured in our study was 1.19 μg/g, a previous study reported a sorghum variety with β-carotene concentrations as high as 3.23 μg/g [20]. In that study, crosses with high carotenoid parents resulted in F2 progeny with β-carotene as high as 3.57 μg/g, suggesting that classical breeding can increase concentrations further. Overall, our findings, along with these previous studies, suggest that sorghum provitamin A carotenoid biofortification is feasible using breeding coupled with modern genomic breeding tools.

In order to ensure continuous genetic gains and trait improvement, genetic diversity is necessary. For carotenoid content in sorghum, however, this might be a limitation, because high carotenoid lines appear to be highly related (Figs. 4A and 5E-G). The tight clustering of countries (Fig. 5E-G) and few countries with high carotenoid lines suggest that there has not been much exchange of germplasm among the countries and most germplasm might not have high carotenoid alleles. Interestingly, here we identified Nigeria, Lebanon, and the United States as the major origins with high carotenoid germplasm both in the SAP/CAP collection and the unexplored germplasm collections. However, it seems that the high carotenoid lines from Lebanon and the United States are from Nigerian origin. In the 1950s, a breeder named O.J. Webster collected yellow endosperm kaura sorghums from Nigeria, which were subsequently used for breeding material in the United States [39]. Some of these kaura lines were then sent by another breeder, R.E. Karper, from the United States to the Arid Land Agricultural Development (ALAD) Program in Lebanon, which eventually became the International Center for Agricultural Research in the Dry Areas (ICARDA) [40, 41]. This relationship between the kaura accessions from the United States and Lebanon explains the close genetic similarity identified in our study between accessions from the United States and Lebanon (Fig. 4A).

Selection for kaura types could also be the underlying driver of the limited genetic diversity and selection signals observed for the high carotenoid lines (Fig. 4B). In Nigeria, kaura types are one of the most common sorghum landraces grown due to their high yield, drought resistance, and grain quality [42]. They also have widely-sought agronomic traits as they are generally of short stature, have large yellow seeds, and are photoperiod insensitive. In the United States, selection for kaura types could also have contributed further to the limited genetic diversity. The first yellow hybrids developed in the United States, using Nigerian germplasm, had stronger root development, improved stay-green, and resistance to charcoal rot [39], which could have led to the incorporation of high carotenoid alleles into multiple pedigrees. Also, among the high carotenoid accessions identified in our study, several of them are listed in GRIN as kaura (durra-caudatums), which could support the indirect selection for carotenoids among the kaura types. All together, these results suggest that kaura types from Nigeria are the main source of high carotenoid alleles and that efforts to increase diversity can focus on them.

Here, we identified 107 accessions from Nigeria, Niger, Ethiopia, Senegal, Sudan and the SAP/CAP collection (Table S5) that have a high GEBV for β-carotene. These accessions need to be phenotyped with HPLC to test the hypothesis that they have high β-carotene concentrations and have potential as donor parents for breeding efforts. It should be noted that if the genetic diversity of the SAP/CAP collection does not fully encompass the range of genetic diversity of the unexplored germplasm, then we may have underestimated some of the predicted values and failed to identify some high carotenoid lines. Importantly, some of the lines hypothesized to have high carotenoids based on GEBV are highly genetically divergent from the high-carotenoid Nigerian germplasm (e.g. Ethiopia and Sudanese lines in Fig. 5, Table S5), suggesting that previously untapped high-carotenoid germplasm exists. It should be noted that since there are so many high carotenoid accessions of the kaura type, it is possible that the signal in the GEBV is related more to the botanical type and geographic origin than high carotenoid concentrations, per se. In the future this can be tested through crosses that break up population structure using germplasm that vary for carotenoid levels. For example, biparental populations could be developed with kaura parental lines crossed with germplasm from other regions. Given that sorghum germplasm does not meet current target values, breeding will be necessary to increase β-carotene concentrations. The high heritabilities for β-carotene [15, 16] suggest that increasing concentrations through breeding is possible. Developing crosses among high β-carotene lines in the SAP/CAP collection as well as those identified by genomic prediction, can provide insights into if there is enough genetic diversity to reach target values. Genomics-assisted breeding via MAS or GS has the potential to accelerate efforts by simplifying selection methods.

### Marker-assisted selection for sorghum carotenoids

MAS could be a viable alternative to select carotenoids in sorghum given that GWAS suggests an oligogenic architecture (Fig. 2 and [15]). MAS for carotenoids has been tested in cassava with a marker linked to the *PSY* gene, which initiates the first committed step in the carotenoid biosynthesis pathway, demonstrating prediction accuracies above 0.8 [43]. MAS has also been implemented in maize biofortification efforts with markers in linkage with the biosynthesis genes *lcyE* [44], *crtRB1* [44–46], *ZEP* [45], and *opaque 2* [47]. For a successful implementation of MAS for sorghum carotenoids, breeder-friendly markers (i.e. convenient and low-cost) with tight linkage and high LD with target alleles must be developed [48].

Chromosome 6 might be a good place to start for sorghum carotenoid marker development due to the high number of associations detected for β-branch carotenoids, where most of the provitamin A carotenoids are synthesized. Four regions of association on chromosome 6 have been identified: 46.7 Mb [15], 50.3–53.5 Mb [16], 57.4 Mb [15] and 47.1 Mb in this study. For β-carotene, associations have been detected in proximity to *phytoene desaturase* (*PDS*, Sobic.006G232600) [15], the second enzyme in the carotenoid biosynthesis pathway. For zeaxanthin, associations have been detected near *zeta-carotene desaturase* (*ZDS*, Sobic.006G177400) [16] and *ZEP* (Sobic.006G097500) [15]. Interestingly, in this study we also identified significant associations near *ZEP* for lutein, zeaxanthin, and β-carotene (Fig. 2, Table S3). These genes have also been associated with natural variation of carotenoids in maize [30]. If no linkage drag is present, the prevalence of associations on chromosome six could mean that positive alleles for multiple genes could be introduced simultaneously, reducing the generations needed. Understanding the allelic diversity of these genes in sorghum germplasm and the expression profiles among high carotenoid germplasm could further demonstrate their potential for utilization in vitamin A biofortification.

The *ZEP* gene in sorghum could be a candidate to initiate such efforts. Based on this and our previous study [15] *ZEP* is a core gene controlling variation in the sorghum β-branch carotenoids, i.e. zeaxanthin and β-carotene. *ZEP* is also a good candidate for breeding efforts though MAS as it seems to have allelic variants with strong correlation with carotenoid concentrations. Marker S06_46717975 is a biallelic allele (A/G) in the *ZEP* coding sequence and is in proximity to S06_47123508, here associated with β-carotene and zeaxanthin. In the SAP/CAP collection, the allele was minor with only 10% of the accessions having the ‘A’ allele. The geographic distribution of the ‘A’ allele also correlates with Nigeria, United States, and Lebanon, the countries that had the highest observed or predicted β-carotene concentrations (Fig. 6). Interestingly, among the unexplored germplasm collections, the top 5% had a more balanced distribution of the A/G variants. One hypothesis is that the top 5% of β-carotene GEBV might capture a wider carotenoid content and diversity than what is present in the SAP/CAP collection and therefore the allele has not been fixed (Table S6). The higher prevalence of countries in the top 5% of GEBV for β-carotene, the lower GEBV when compared to the SAP/CAP collection (Fig. S1), and the more sparsely grouped cluster (Fig. 5G) here observed supports this hypothesis. However, we hypothesize that due to the high correlation of allelic variant and carotenoid content observed and predicted, marker S06_46717975 could be used for MAS and to identify potential donor lines. Further germplasm evaluations are needed to assess this marker's predictive ability.

Finally, it should be noted that as with previous studies, GWAS here identified a total of 27 regions of association, but only two of those regions were near known carotenoid pathway genes. This demonstrates that there are still many unknown genes involved in carotenoid variation, which are perhaps regulatory pathway controls or unidentified homologues of carotenoid biosynthesis or degradation genes. Further studies, such as transcriptomics, are needed to help find the causal genes in linkage with markers identified through GWAS.

### Genomic selection for sorghum carotenoids

Genomic selection is an alternative to MAS that is increasingly used for complex traits as genotypic costs decrease. For quality traits GS could potentially reduce the cost compared to phenotyping, and reduce the need for specialized equipment and training. In wheat, GS has been proven to be superior for quality traits over MAS as it allows for the selection for both small and large effect loci [49–51]. GS for carotenoids has yet to be implemented in breeding programs, but it has been tested in cassava [52] and maize [53]. Here we report the first study on genomic prediction for sorghum carotenoids. Genomic predictions are designed to capture polygenic variance and allow for selection on complex traits. GS accuracy and efficiency is dependent on several factors, particularly heritability of the trait, because it often directly translates into the prediction accuracy. The high heritability estimates (0.78, Table 1) and prediction accuracy (0.67, Table 2) here obtained for β-carotene would suggest that there is a polygenic component to sorghum carotenoids and GS can be an efficient method for biofortification. One hypothesis that explains why we see evidence for both oligogenic and polygenic variation is that sorghum carotenoids are omnigenic traits, in which a small number of core genes directly regulate carotenoids and a large number of peripheral genes that are expressed in the grain indirectly regulate carotenoids [54]. This hypothesis could be tested with a genome-wide expression study in high carotenoid germplasm.

Despite its potential, there are several factors to consider in GS for sorghum carotenoids. First, the results of this study suggest that most countries' germplasm currently lack enough phenotypic and genotypic diversity for sorghum carotenoids. This suggests that the next step for provitamin A biofortification would be to introduce high carotenoid alleles into these breeding programs via pre-breeding. Given that oligogenic variation for grain carotenoids exists, this initial introduction of alleles could be accomplished with MAS. Second, even though GS has the potential to reduce cost of phenotyping, simulation studies suggest that depending on population size, genotyping costs must be under $15 (U.S. Dollars) to be more cost-effective than simple phenotypic selection [55]. This genotyping cost can make GS unrealistic for breeding programs in developing countries, which would be the ones to benefit the most from a biofortified sorghum, as it is estimated that genotyping several hundred SNP markers remains at $14 (U.S. Dollars) [56].

Lastly, incorporation of a GS scheme for a young breeding program can be very challenging. GS has the biggest potential for genetic gain per unit of time when breeding cycles are closed rapidly and effectively [55–57]. However, many breeding programs in developing countries have slow breeding cycles with recycling improved lines as a parent often taking well over 10 years [58]. Therefore, under these scenarios we suggest the direction for biofortification breeding will be to first introduce major genes through MAS in breeding programs. After the introduction of these alleles, and as genotyping cost continues to decrease, MAS in tandem with GS can then be used for continuous improvement. If carotenoid variation in sorghum is in fact both oligogenic and polygenic, then the incorporation of MAS, GS, and rapid breeding cycles could substantially increase β-carotene to target values and ensure continuous genetic gains.

## Conclusions

In this study we evaluated carotenoid concentrations in SAP/CAP collection, identifying the accessions with the highest β-carotene concentrations. Also, it was established that current concentrations of β-carotene are low and current known high β-carotene germplasm has a narrow genetic diversity. We used the SAP/CAP collection as a training population to predict the genetic merit or GEBV via genomic prediction for unexplored germplasm. Based on GEBV, we present 107 accessions with the highest predicted concentrations for β-carotene that potentially represent novel genetic variation for the trait. Finally, we proposed that MAS should be initially used to introduce high carotenoid alleles like S06_46717975 inside the *ZEP* gene into breeding programs followed by GS for continuous improvement.

## Methods

### Plant material

Grain carotenoid concentration was evaluated for a total of 446 sorghum accessions, which included 316 from the sorghum association panel (SAP) [59] and 130 accessions from the carotenoid panel (CAP), a set of accessions chosen for presence of yellow endosperm and/or yellow grain [15, 60]. The two panels were grown, selfed, and harvested by the authors at Kansas State University Agronomy North Farm in Manhattan, Kansas with a randomized complete block design with two replications during the summer of 2019. At maturity, grain was harvested, dried and stored at -80 °C until carotenoid quantification.

### Carotenoid quantification

Carotenoid extractions were performed following a modified solid phase method [61]. All steps of the extraction were carried out under yellow light to avoid photodegradation. Briefly, approximately 5 seeds were ground to flour using a Bead Ruptor Elite (Omni International, Kennesaw, GA) and 20 mg of the sorghum flour were transferred to a 1.5 mL eppendorf tube. Next, 20 mg of ascorbic acid and 400 μl of absolute ethanol with a 1 mg/mL concentration of butylated hydroxytoluene (BHT) were added. The tubes were vortexed for 1 min and placed in an 80 °C water bath for 5 min. Following the incubation, 20 μl of a solution of potassium hydroxide (80% w/v, in water) was added and tubes vortexed for 1 min. Next, samples were returned to the water bath for 15 min and mixed every 5 min. Samples were then brought to room temperature and centrifuged for 5 min at 1800 rcf. Supernatant was transferred to a new 1.5 mL eppendorf tube. An additional 400 μl of absolute ethanol with 1 mg/mL of BHT was added to the residue, vortexed for 1 min and centrifuged for 5 min at 1800 rcf. The supernatant was combined with the above extract, vortexed for 30 s and centrifuged for 5 min at 5000 rcf. The supernatant was then transferred to a new 1.5 mL eppendorf tube and evaporated to dryness with a gentle N_2_ stream at room temperature. Finally, the residue was reconstituted in 100 μl of Methanol:Ethyl Acetate (1:1) and centrifuge for 5 min at 5000 rcf. An aliquot of 40 μl of the clear supernatant was utilized for the HPLC analysis. Resolution of lutein, zeaxanthin, β-carotene, α-carotene and β-cryptoxanthin was conducted using an Agilent 1290 Infinity UHPLC with Eclipse Plus C18 column (Agilent Technologies, Santa Clara, California, U.S).

### Statistical analysis of carotenoids and heritability estimation

Concentrations for lutein, zeaxanthin and β-carotene were analyzed with ASReml-R package [62], which accounts for missing data. For the three carotenoids, we implemented a randomized complete block design model with genotype and block as random effects. The gamma parameterization with a maximum iteration number of 100 was used for the analysis. Best linear unbiased predictors (BLUPs) were obtained as predictors of genetic merit for lutein, zeaxanthin and β-carotene and were used for subsequent analysis. Broad sense heritability on an entry-mean was also calculated for lutein, zeaxanthin and β-carotene as followed:

$${H}^{2}=\frac{{\sigma }_{Genotype}^{2}}{{\sigma }_{Phenotype}^{2}}= \frac{{\sigma }_{Genotype}^{2}}{{\sigma }_{Genotype}^{2}+\frac{{\sigma }_{error}^{2}}{r}}$$

where H^2^ represents the broad sense heritability on a entry-mean basis, ${\sigma }_{Genotype}^{2}$ represents the genotypic variance, ${\sigma }_{Phenotype}^{2}$ the phenotypic variance and ${\sigma }_{error}^{2}$ the residual or error variance, and r represents the blocks. Pearson correlations were calculated between carotenoid pairs for lutein, zeaxanthin, and β-carotene.

### Genome-wide association study

The genetic architecture and the genomic regions underlying carotenoid variation in sorghum grain were investigated through a genome-wide association study (GWAS) implemented in GAPIT [63], version “2022.4.16, GAPIT 3.1″. The genotype information for the sorghum association panel and the carotenoid panel was obtained from previous studies [15, 64]. The SNP datasets are available for download from the Dryad Data Repository (doi:10.5061/dryad.63h8fd4). After filtering the single nucleotide polymorphism (SNP) data set (Sorghum bicolor v3.1 genome version) by a minimum allele frequency of 0.05, 348,181 biallelic SNPs remained. A total of 345 accessions for the SAP/CAP collection had both genotype information and BLUP estimates for lutein, β-carotene, and zeaxanthin. A mixed linear model (MLM) (model = ”MLM”) was used with a marker derived kinship and ten principal components (PCA.total = 10) to control for relationship and population structure, respectively. To account for multiple comparisons, the Bonferroni correction with *P* = 0.05 was used to identify significant SNPs. Significant associations were compared with candidate genes that are annotated as enzymes involved in the carotenoid pathway in Phytozome or that have been identified in other carotenoid association studies (Table S7).

### Diversity of high carotenoids lines in the SAP/CAP global collection

Genetic diversity among the high carotenoid lines identified was examined. We prioritized assessing the genetic diversity among the accessions with the highest β-carotene BLUP estimates, because β-carotene is the most abundant provitamin A carotenoid in sorghum. Accessions were ranked as top 5% and bottom 5% based on their BLUP estimate for β-carotene. A marker-derived additive relationship matrix or kinship, was estimated with the ‘A.mat’ function in the rrBLUP R package [65]. The eigenvalues for the first two principal components were estimated with R function ‘eigen’ for the additive relationship matrix and the grouping of the top 5% was examined. Genetic diversity of regions associated with β-carotene variation was determined using a window of 1 megabase (Mb) upstream and downstream of significant SNPs identified by GWAS in proximity to a priori candidate genes. The linkage disequilibrium (LD) for the region was calculated with rTASSEL [66] for all the SNPs within the 2 Mb window and setting heterozygous as “missing” (Fig. S3). Nucleotide diversity (π) per base pair was calculated with rTASSEL [66] using a step size of 100 and a window size of 500.

### Genotype from unexplored germplasm collections

Publicly available genotype data from unexplored germplasm collections were gathered. Accessions and their corresponding genotype information from Ethiopia [67], Haiti [36], Niger [68], Nigeria [64, 69, 70], Senegal [71] and Sudan [37] were obtained from published data or by contacting the authors. Common SNPs between the unexplored germplasm collections and the SAP/CAP global collection that had at least 80% of the data present were identified. Missing SNP data were then imputed using Beagle [72] with the default parameters. To assess the genetic relationships among the accessions in the unexplored germplasm and the SAP/CAP collection a realized additive relationship matrix was calculated first using the ‘A.mat’ function in rrBLUP R package [65]. The additive relationship matrix was then used to perform a principal component analysis (PCA) in R.

### Genomic prediction of GEBV for carotenoids in unexplored germplasm

Predictions of the genomic estimated breeding values (GEBV) and genomic heritability were conducted using the genomic data from unexplored germplasm, representing country collections of Ethiopia, Haiti, Niger, Nigeria, Senegal, Sudan, and the SAP/CAP collection. The accessions in the SAP/CAP collection for which we had genotype information and BLUPs estimates for lutein, zeaxanthin, and β-carotene were used as the training population (n = 345). GEBV were estimated for lutein, zeaxanthin and β-carotene using the G-BLUP model with the additive relationship matrix or kinship as implemented in the rrBLUP package in R [65]. A fivefold cross validation approach was used for each carotenoid to determine prediction accuracy. The cross validation was repeated for 100 cycles. Genomic heritability for lutein, zeaxanthin and β-carotene was estimated during each k-fold and cycle. Prediction accuracy was also estimated by calculating the correlation between the genomic prediction and the validation values divided by square root of heritability [73]. The unexplored germplasm was ranked in the top 5% for lutein, zeaxanthin and β-carotene based on the GEBV estimates. A marker-derived kinship was estimated with the ‘A.mat’ function in the rrBLUP R package [65] for the unexplored germplasm. The eigenvalues for the first two principal components were estimated with R function ‘eigen’ for the additive relationship matrix and the grouping of the top 5% for each of the carotenoids was examined. Additionally, the distribution of the GEBVs for β-carotene was evaluated by country. Lastly, we compared the GEBV for the three carotenoids in the SAP/CAP collection and unexplored germplasm collections.

### Allelic diversity and geographic distribution of ZEP allele

Distribution of the *ZEP* allele for the SAP/CAP collection and the unexplored germplasm collections was examined using country of origins and the allelic classes for the SNP S06_46717975. Countries that had less than 3 accessions were discarded from the analysis. We also aggregated the allelic variants present in the high β-carotene accessions from the SAP/CAP collection and the unexplored germplasm collections, as defined by the top 5% of BLUP or GEBV for β-carotene.

## Supplementary Information

**Additional file 1: Table S1.** Raw carotenoid concentrations in SAP/CAP. **Table S2.** BLUPs for carotenoids in the SAP/CAP. **Table S3.** Carotenoids marker-trait associations in the SAP/CAP. **Table S4.** Carotenoid GEBV in SAP/CAP and unexplored germplasm. **Table S5.** Top 5% β-carotene GEBV in unexplored germplasm. **Table S6.** Allelic distribution of ZEP SNP S06_46717975. **Table S7.** A priori carotenoid candidate genes. **Table S8.** Common name, endosperm color, kernel color, race, and working group of evaluated accessions.

**Additional file 2: Fig. S1.** Comparison of GEBV for lutein, zeaxanthin, and β-carotene for the SAP/CAP global collection and the unexplored germplasm collections. Boxplot of GEBV for A) lutein; B) zeaxanthin; and C) β-carotene.

**Additional file 3: Fig. S2.** PCA of genetic relationship between SAP/CAP and unexplored germplasm.

**Additional file 4: Fig. S3. **LD for 1 Mb region upstream and downstream of marker S06_47123508.

## Data Availability

The variant data for this study have been deposited in the European. Variation Archive (EVA) at EMBL-EBI under accession number PRJEB60406. https://www.ebi.ac.uk/eva/?evastudy=PRJEB60406 Original publication with the genotype data can be found in the following citations: • Ethiopia germplasm [67] • Haiti germplasm [36] • Niger germplasm [68] • Nigeria germplasm [64, 69, 70] • Senegal germplasm [71] • Sudan germplasm [37] • SAP/CAP germplasm [64]

## Abbreviations

BLUP: Best linear unbiased prediction
CAP: Carotenoid panel
FDR: False discovery rate
GWAS: Genome-wide association study
GEBV: Genomic estimated breeding value
GGPS: Geranylgeranyl diphosphate synthase
GS: Genomic selection
HPLC: High-performance liquid chromatography
MAS: Marker-assisted selection
Mb: Megabase
MLM: Mixed linear model
*PDS*: *Phytoene desaturase*
*PSY*: *Phytoene synthase*
PCA: Principal component analysis
SAP: Sorghum association panel
SNP: Single nucleotide polymorphism
*ZEP*: *Zeaxanthin epoxidase*
