# Supplementary figures and images for: Characterization of grain carotenoids in global sorghum germplasm to guide genomics-assisted breeding strategies
Source: BMC Plant Biol. 2023 Mar 28;23:165. doi: 10.1186/s12870-023-04176-0 (PMC10045421; doi:10.1186/s12870-023-04176-0)

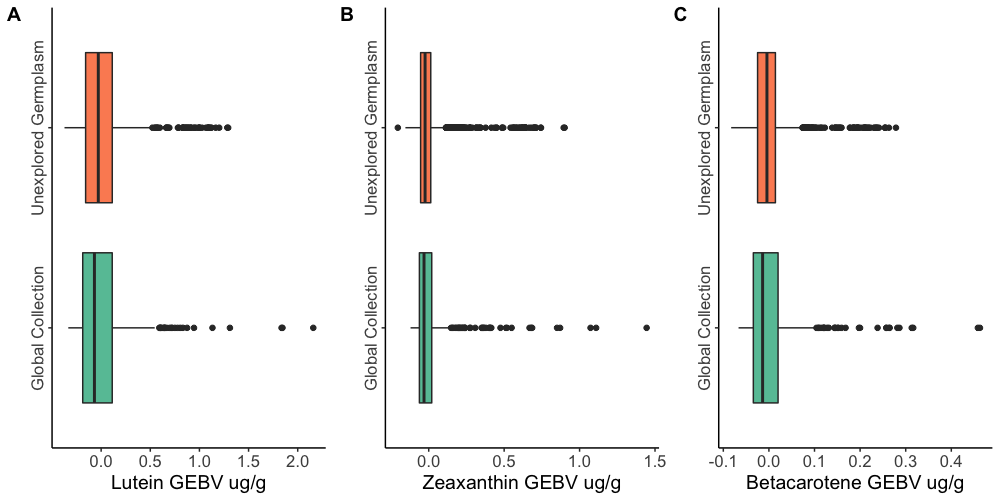

Supplement: Supplementary file 2 — Additional file 2: Fig. S1. Comparison of GEBV for lutein, zeaxanthin, and β-carotene for the SAP/CAP global collection and the unexplored germplasm collections. Boxplot of GEBV for A) lutein; B) zeaxanthin; and C) β-carotene. [file 12870_2023_4176_MOESM2_ESM.png]

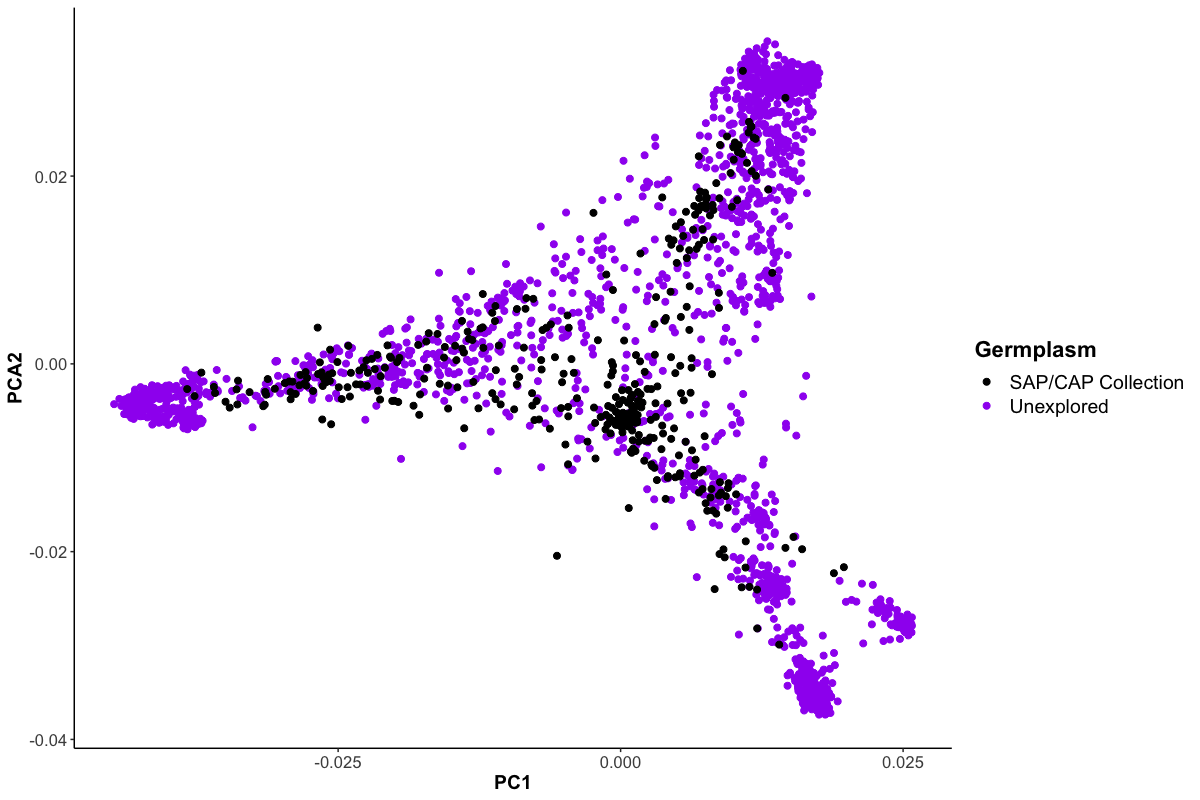

Supplement: Supplementary file 3 — Additional file 3: Fig. S2. PCA of genetic relationship between SAP/CAP and unexplored germplasm. [file 12870_2023_4176_MOESM3_ESM.png]

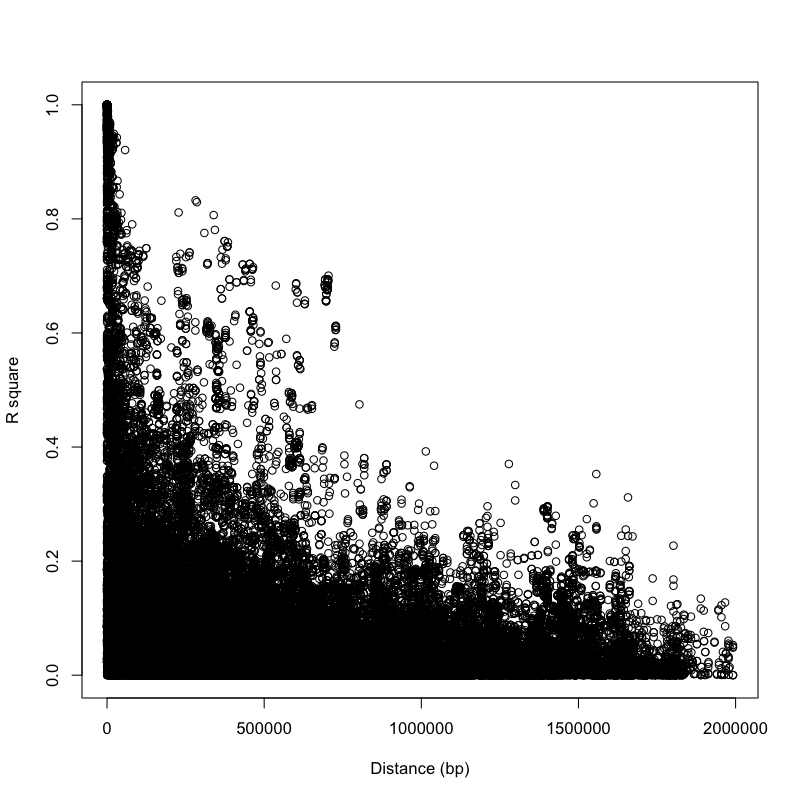

Supplement: Supplementary file 4 — Additional file 4: Fig. S3. LD for 1 Mb region upstream and downstream of marker S06_47123508. [file 12870_2023_4176_MOESM4_ESM.png]
